# Combined Treatment with Troglitazone and Lovastatin Inhibited Epidermal Growth Factor-Induced Migration through the Downregulation of Cysteine-Rich Protein 61 in Human Anaplastic Thyroid Cancer Cells

**DOI:** 10.1371/journal.pone.0118674

**Published:** 2015-03-05

**Authors:** Li-Han Chin, Sung-Po Hsu, Wen-Bin Zhong, Yu-Chih Liang

**Affiliations:** 1 Graduate Institute of Medical Sciences, College of Medicine, Taipei Medical University, Taipei, Taiwan; 2 Department of Physiology, College of Medicine, Taipei Medical University, Taipei, Taiwan; 3 School of Medical Laboratory Science and Biotechnology, College of Medical Science and Technology, Taipei Medical University, Taipei, Taiwan; 4 Traditional Herbal Medicine Research Center, Taipei Medical University, Taipei, Taiwan; National Cheng Kung University, TAIWAN

## Abstract

Our previous studies have demonstrated that epidermal growth factor (EGF) can induce cell migration through the induction of cysteine-rich protein 61 (Cyr61) in human anaplastic thyroid cancer (ATC) cells. The aim of the present study was to determine the inhibitory effects of combined treatment with the peroxisome proliferator-activated receptor-γ (PPARγ) ligand troglitazone and the cholesterol-lowering drug lovastatin at clinically achievable concentrations on ATC cell migration. Combined treatment with 5 μM troglitazone and 1 μM lovastatin exhibited no cytotoxicity but significantly inhibited EGF-induced migration, as determined using wound healing and Boyden chamber assays. Cotreatment with troglitazone and lovastatin altered the epithelial-to-mesenchymal-transition (EMT) -related marker gene expression of the cells; specifically, E-cadherin expression increased and vimentin expression decreased. In addition, cotreatment reduced the number of filopodia, which are believed to be involved in migration, and significantly inhibited EGF-induced Cyr61 mRNA and protein expression as well as Cyr61 secretion. Moreover, the phosphorylation levels of 2 crucial signal molecules for EGF-induced Cyr61 expression, the cAMP response element-binding protein (CREB) and extracellular signal-regulated kinase (ERK), were decreased in cells cotreated with troglitazone and lovastatin. Performing a transient transfection assay revealed that the combined treatment significantly suppressed Cyr61 promoter activity. These results suggest that combined treatment with low doses of troglitazone and lovastatin effectively inhibits ATC cell migration and may serve as a novel therapeutic strategy for metastatic ATC.

## Introduction

Anaplastic thyroid cancer (ATC) is among the most aggressive malignancies with extremely short survival and poor prognosis. ATC accounts for approximately 5% to 15% of primary malignant thyroid tumors that are resistant to surgery, radiotherapy, and chemotherapy [1, 2]. No curative options are available for patients with ATC, and the poor prognosis is attributed to its unlimited growth and invasive migration. Therefore, identifying new therapeutic strategies is critical for ATC management.

The epidermal growth factor receptor (EGFR), a receptor tyrosine kinase, belongs to the HER/ErbB, protein family. Epidermal growth factor (EGF), a ligand of the EGFR, can bind to and activate the EGFR and then transduce the proliferation and survival signals primarily mediated by both mitogen-activated protein kinase (MAPK) and phosphatidylinositol-3′ kinase (PI_3_K) [3]. Increased EGFR expression is considered a negative prognostic factor for various types of cancer, such as bladder [4] and breast cancers [5]. A preclinical study indicated that EGF is involved in the proliferation and migration of follicular and papillary thyroid cancer [6]. In addition, EGF or EGFR overexpression was observed in most thyroid cancer cells, including ATC cells [7]. In addition, increased EGF expression is associated with poor prognosis in patients with metastatic thyroid cancer [7]. Moreover, a study indicated that the EGFR is a novel therapeutic target for treating patients with ATC [8].

The CCN family of growth regulators comprises cysteine-rich protein 61 (Cyr61, also known as CCN1), connective tissue growth factor (CTGF, also known as CCN2), and nephroblastoma overexpressed (Nov, also known as CCN3) [9]. Cyr61 is secretory protein involved in the regulation of cell adhesion, DNA synthesis, angiogenesis, cell survival, and migration [10, 11].

Thiazolidinediones (TZDs) are synthetic peroxisome proliferator-activated receptor-γ (PPARγ) agonists that have been widely used in treating type 2 diabetes and can inhibit cellular growth through PPARγ-dependent or -independent pathways. Studies have shown that PPARγ activation either inhibits cell proliferation or induces apoptosis in various types of cancer [12, 13]. Troglitazone, a member of the TZD family, has been reported to induce apoptosis and inhibit cell migration and proliferation in numerous types of human cancer cell, including thyroid cancer [14, 15]. Lovastatin, a competitive inhibitor of 3-hydroxy-3-methylglutaryl coenzyme A (HMG-CoA) reductase, inhibits the conversion of mevalonate from HMG-CoA. Clinically, it has been used to reduce cholesterol levels in hypercholesterolemia. In addition, lovastatin serves several other biological functions, such as the inhibition of cell proliferation, adhesion, and migration in various types of cancer cell [16, 17].

Our previous study demonstrated that lovastatin can induce apoptosis and repress cell migration in ATC cells by inhibiting the Rho/ROCK signaling pathways [18]. In this study, troglitazone and lovastatin were combined to increase the efficacy of lovastatin in treating ATC. The aim of this study was to elucidate the combined effects of troglitazone and lovastatin on EGF-induced migration in addition to the underlying molecular mechanisms in ATC cells.

## Materials and Methods

### Reagents

Troglitazone was purchased from Sigma-Aldrich (St. Louis, MO, USA), and lovastatin was provided by the Standard Chemical & Pharmaceutical Co. (Tainan, Taiwan). Recombinant human EGF was purchased from R&D Systems (Minneapolis, MN, USA). EGFR and phospho-EGFR antibodies were purchased from GeneTex (San Antonio, TX, USA). Polyclonal antibodies against anti-Cyr61 antibodies and phospho-extracellular signal-regulated kinase (ERK) antibodies were purchased from Santa Cruz Biotechnology (Santa Cruz, CA, USA). Monoclonal antibodies against phospho-cAMP response element-binding protein (CREB) (Ser133) were purchased from Millipore (Bedford, MA, USA). Polyclonal antibodies against vimentin and E-cadherin were purchased from Cell Signaling Technology (Beverly, MA, USA). Antibodies against total ERK were purchased from the Sigma Chemical Co. (St. Louis, MO, USA).

### Cell Culture

SW1736 and 8305C human ATC cells were obtained from Carl-Henrik Heldin (Ludwig Institute for Cancer Research, Uppsala University, Uppsala, Sweden) and Paolo Vigneri (Department of Biomedical Sciences, Section of General Pathology, University of Catania, Italy), respectively [19]. Both the SW1736 and 8305C cells were cultured in an RPMI 1640 medium (Sigma Chemical Co.) supplemented with 5% fetal-bovine serum (FBS; Life Technologies, Grand Island, NY, USA) and antibiotics (100 U/mL of penicillin and 100 mg/mL of streptomycin) at 37°C in a 5% CO_2_ incubator.

WI-38 human lung fibroblast cells were obtained from the American Type Culture Collection (Manassas, VA, USA) and cultured in a minimum essential medium (Sigma Chemical Co.) supplemented with 5% FBS and antibiotics (100 U/mL of penicillin and 100 mg/mL of streptomycin) at 37°C in a 5% CO_2_ incubator.

### Cell Viability Assay

The drug-treated cells were fixed in 4% paraformaldehyde and stained with 0.1% crystal violet dye. Subsequently, this dye was eluted using 40% acetic acid, and the absorption was determined using a microtiter plate reader (BioTek, μQuant, Toronto, Canada) at OD_570_ nm.

### Wound Healing Assay

The cells were seeded in a Culture-Insert 24 to create a cell-free gap of 500 μm. After the Culture Insert was removed, the cells were incubated with troglitazone and lovastatin for 0.5 h in the presence or absence of EGF or Cyr61 for 7 or 9 h, respectively. The migrated cells were counted under an optical microscope.

### Transwell Migration Assay

Cell migration was assayed using the polycarbonate membranes (8-μm pore size) of transwell culture chambers. After migration, the cells on the upper surface of the membrane were removed using a cotton swab, and the cells on the lower surface of the membrane were fixed with 4% paraformaldehyde and then stained with 0.1% crystal violet. The migrated cells were counted in 5 distinct fields and photographed under a light microscope.

### Fluorescence Microscopy

The drug-treated cells were fixed in 4% paraformaldehyde; permeabilized through overnight treatment with 0.2% Triton X-100, 0.05% Tween 20, and 0.3% BSA in PBS; and stained with rhodamine phalloidin (ICN Immunobiologicals, Costa Mesa, CA, USA) to detect actin polymerization and with 4',6-diamidino-2-phenylindole (DAPI) to detect the nuclei. Fluorescent images were acquired using a CM350 CCD camera (Applied Precision, Issaquah, WA, USA) and the TCS SP5 confocal spectral microscope imaging system software (Leica, Wetzlar, Germany) and processed using the Photoshop CS5 EXTENDED Version 12.0.4 software (Adobe System, San Diego, CA, USA).

### RNA Isolation, Semiquantitative Reverse-Transcription Polymerase Chain Reaction (RT-PCR), and Real-Time RT-PCR

The total RNA was isolated from cultures by directly lysing the cells in a Trizol reagent (Invitrogen, Carlsbad, CA, USA) according to the manufacturer’s instructions. Subsequently, 2 μg of the total RNA were reverse transcribed to synthesize complementary (c) DNA by using the SuperScript III First-Strand Synthesis System for the reverse-transcription polymerase chain reaction (RT-PCR) (Invitrogen).

The mRNA expression levels were measured using the LightCycler 480 and SYBR Green I Master reagent (Roche Applied Science, Germany). The thermal cycling conditions included an initial 20-s holding period at 95°C, followed by a 3-step PCR program repeated for 40 cycles: 3 s at 95°C, 30 s at 60°C, and 30 s at 60°C. The following oligonucleotide primers were used: E-cadherin forward 5′-CCTGGGACTCCACCTACAGA-3′, reverse 5′-GGATGACACAGCGTGAGAGA-3′; vimentin forward 5′-CCCTCACCTGTGAAGTGGAT-3′, reverse 5′-TCCAGCAGCTTCCTGTAGGT-3′; Cyr61 forward 5′-ACTTCATGGTCCCAGTGCTC-3′, reverse 5′-AAATCCGGGTTTCTTTCACA-3′; GAPDH forward 5′-GAAGGTGAAGGTCGGAGTC-3′, reverse 5′-GGTGGAATCATATTGGAACATGTAA-3′.

### Measurement of the Cyr61 Concentration

The cell culture media were collected and centrifuged at 3000 rpm for 1 min at 4°C. The supernatants were collected, and their Cyr61 concentrations were determined using the ELISA kit (Cusabio Biotech and Adipobioscience, Shanghai, China) according to the manufacturer’s instructions.

### Western Blot Analysis

The total cellular proteins were collected using the PRO-PREP Protein Extraction Solution (iNtRON BIOTECHNOLOGY, Seongnam, Korea). The protein samples (30–50 μg) were subjected to 8% to 10% sodium dodecyl sulfate-polyacrylamide gel electrophoresis and then transferred to Hybond-P polyvinylidine difluoride membranes (Jackson ImmunoResearch Laboratories, West Grove, PA, USA). The primary antibodies were blotted overnight at 4°C, and the horseradish peroxidase-conjugated secondary antibodies were incubated at room temperature for 1 h. The protein bands were visualized using an Enhanced Chemiluminescence (ECL) kit with Fuji Super RX film and were quantified through densitometry by using Image-Pro software.

### Plasmid Transfection and Reporter Luciferase Activity Assay

The human Cyr61 reporter plasmid was provided by Dr. N. Schütze (University of Wurzburg, Wurzburg, Germany) and contained the human Cyr61-gene promoter region −429-+1 and luciferase gene. The cells were seeded in 24-well plates and transiently transfected with the Cyr61 reporter plasmid and phRL-TK plasmid (Promega) as internal controls. Following transfection, the drug-treated cells were collected to detect the luciferase activity by using a dual luciferase assay kit (Promega) in a TD-20/20 luminometer (Turner Designs, Sunnyvale, CA, USA).

### Statistical Analysis

All data were expressed as the mean ± standard error of the mean (SEM). The data were compared using a Student’s *t* test. A *p* value < 0.05 was considered statistically significant.

## Results

### Combined Treatment with Troglitazone and Lovastatin Inhibited EGF-Induced Migration

**Fig 1:**
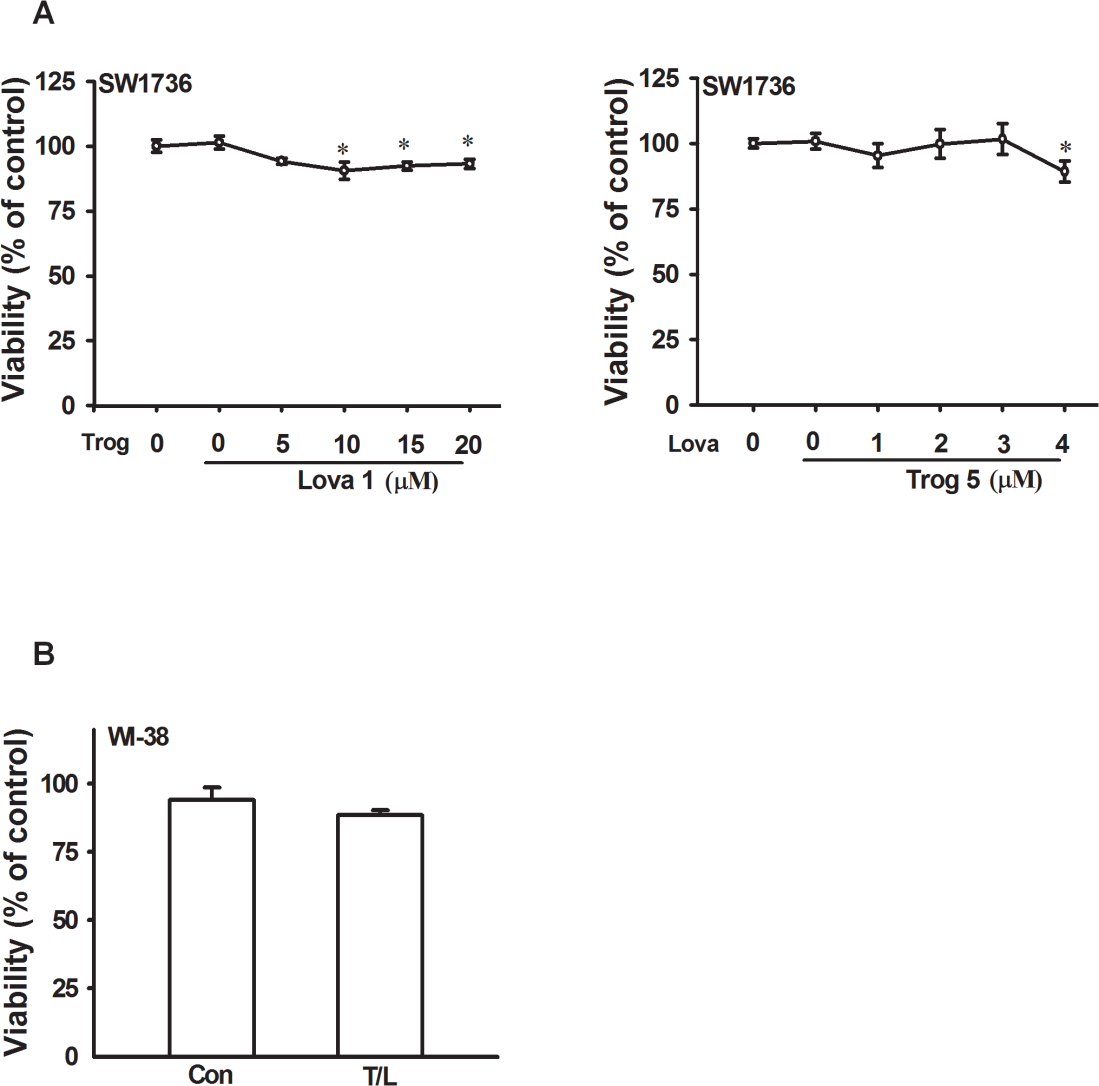
Effects of troglitazone and lovastatin on cell viability in ATC and fibroblast cells. **A.** SW1736 human ATC cells were treated with the indicated concentrations of troglitazone and lovastatin for 24 h. **B.** WI-38 human fibroblast cells were cotreated with troglitazone (5 μM) and lovastatin (1 μM) for 24 h. Cell viability was determined using the crystal violet assay. The data are presented as the mean ± SEM of 3 independent experiments. *, *p* < 0.05, compared with the control group. Con, control group; Trog, troglitazone; Lova, lovastatin; and T/L, combined treatment with troglitazone and lovastatin.

To determine the concentrations of troglitazone and lovastatin that can inhibit cell migration without affecting cell viability, we used the human ATC cell line SW1736 and human fibroblast cell line WI-38. These cells were treated with various concentrations of troglitazone and lovastatin for 24 h, and the cell viability was determined using crystal violet staining. As shown in Fig. 1A, less than 10 μM troglitazone combined with 1 μM lovastatin did not affect the viability of the SW1736 cells. Next, the SW1736 cells were treated with 5 μM troglitazone combined with 1 to 4 μM lovastatin. The results indicated that 5 μM troglitazone combined with less than 4 μM lovastatin did not cause cell death in the ATC cells. Therefore, the cytotoxic effects of 5 μM troglitazone combined with 1 μM lovastatin on the WI-38 human fibroblast cells were evaluated. As shown in Fig. 1B, 5 μM troglitazone combined with 1 μM lovastatin also did not affect the viability of the WI-38 human fibroblast cells. These results suggested that combined treatment with 5 μM troglitazone and 1 μM of lovastatin did not affect the cell viability of either the SW1736 or WI-38 cells; thus, these concentrations were used in the subsequent experiments.

**Fig 2:**
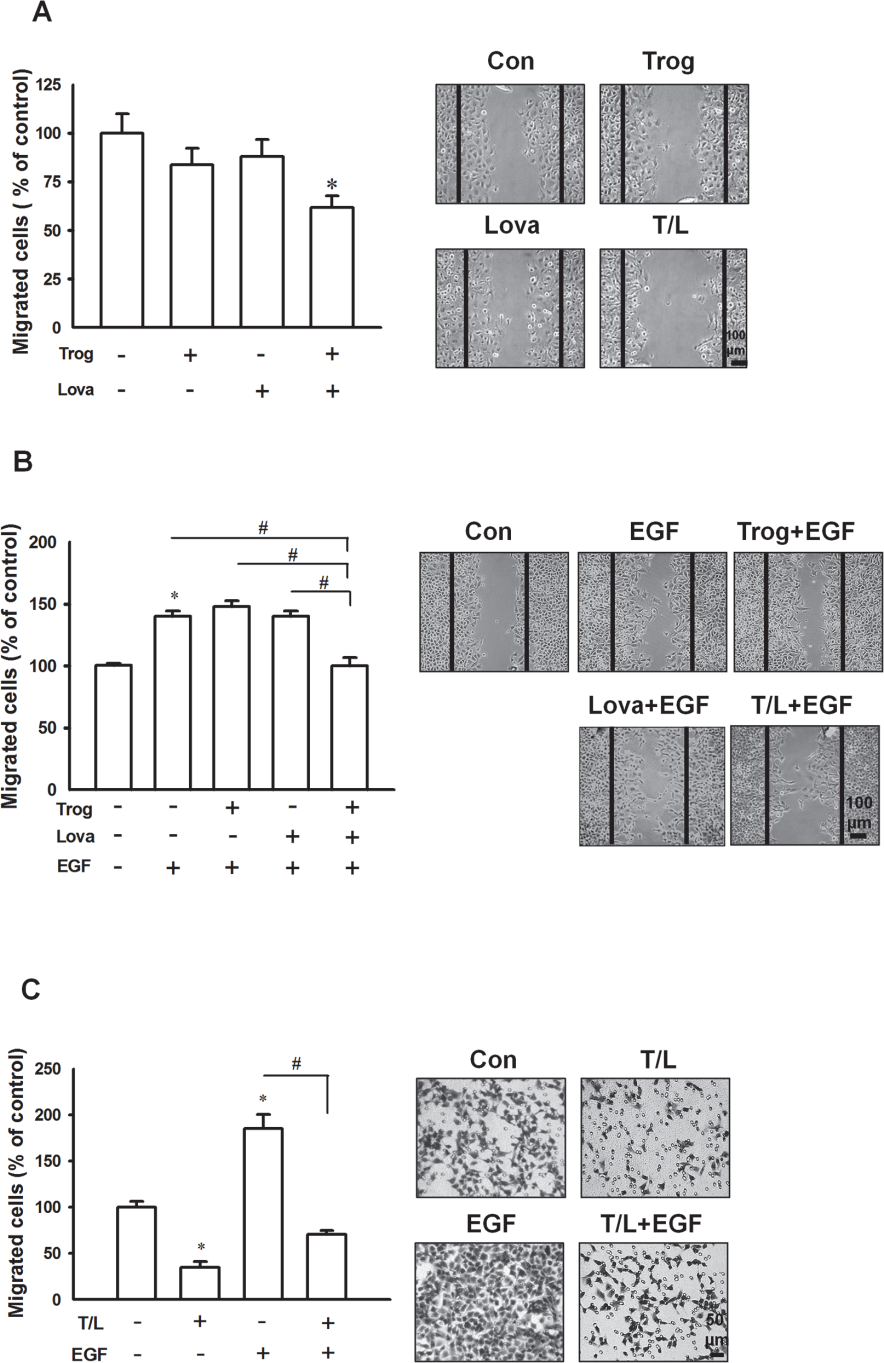
Effects of combined treatment with troglitazone and lovastatin on EGF-induced cell migration in ATC cells. **A.** SW1736 cells were treated with troglitazone (5 μM) and/or lovastatin (1 μM) for 7 h, and the migrated cells were detected using a wound healing assay. **B.** The SW1736 cells were preincubated with troglitazone (5 μM) and/or lovastatin (1 μM) for 0.5 h before EGF treatment. After 7 h, the migrated cells were detected using the wound healing assay. **C.** The SW1736 cells were cotreated with troglitazone (5 μM) and lovastatin (1 μM) for 4 h, and the cells were then seeded in the upper Transwell chamber and EGF (20 ng/mL) was added to the lower chamber as a chemoattractant for cell migration. After 18 h of incubation, the transmigrated cells were stained and counted. All data are presented as the mean ± SE of 3 independent experiments. *p < 0.05, compared with the control group; ^#^, *p* < 0.05. Con, control group; Trog, troglitazone; Lova, lovastatin; and T/L, combined treatment with troglitazone and lovastatin.

We observed that troglitazone combined with lovastatin did not affect cell viability but altered the cell morphology, causing the cells to exhibit epithelial characteristics. To investigate whether combined treatment with troglitazone and lovastatin affected cell migration, a wound healing assay and Boyden chamber model were used. As shown in Fig. 2A and 2B, the combined treatment significantly inhibited cell migration in the cells with or without EGF, but these effects were not observed when either troglitazone or lovastatin were used alone. Moreover, the combined treatment significantly inhibited EGF-induced cell migration in the Boyden chamber (Fig. 2C). In addition to SW1736, another ATC cell line, 8305C, was subjected to the aforementioned combined treatment to examine its antimigration effects. The results revealed that the combined treatment completely inhibited EGF-induced cell migration in these cells (S1 Fig.). These results suggested that the combined treatment inhibits cell migration without affecting the viability of ATC cells.

### Combined Treatment with Troglitazone and Lovastatin Inhibited EGF-Induced Cytoskeletal Reorganization and Altered Epithelial-to-Mesenchymal-Transition Related Marker Protein Expression

**Fig 3:**
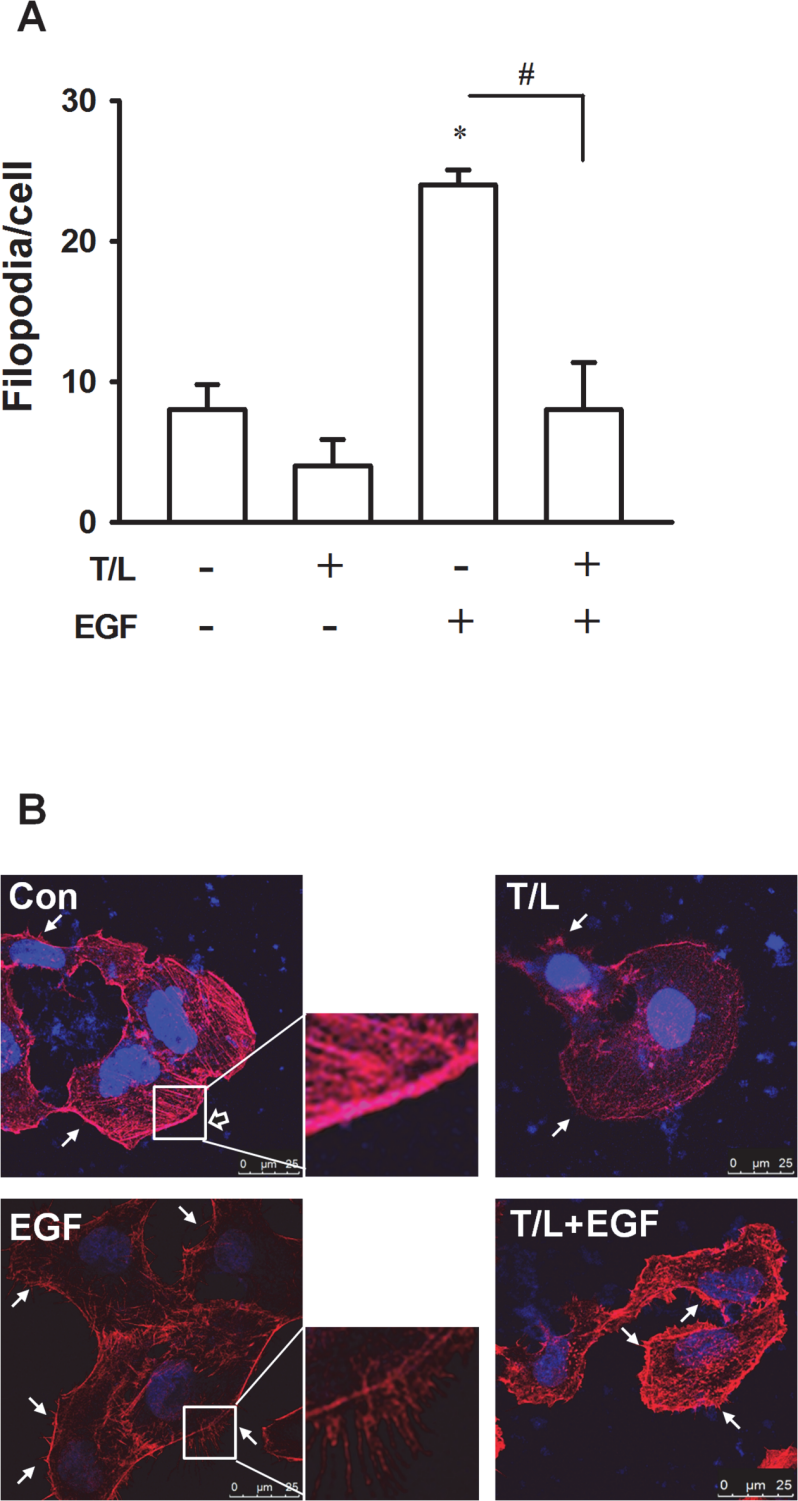
Effects of combined treatment with troglitazone and lovastatin on EGF-induced stress-fiber formation in ATC cells. **A–B.** SW1736 cells were cotreated with troglitazone (5 μM) and lovastatin (1 μM) for 3.5 h and subsequently treated with EGF (10 ng/mL) for 1 h. The cells were fixed, and immunocytochemistry staining was performed using phalloidin (red) to detect actin polymerization and DAPI staining was performed to detect nuclei (blue). **A.** The average number of filopodia per cell was determined by counting 30 cells. **B.** The figure depicts representative images. The arrows indicate the membrane ruffles and filopodia, and the representative regular membrane (open arrow) and filopodia (closed arrow) are shown. Con, control group and T/L, combined treatment with troglitazone and lovastatin.

**Fig 4:**
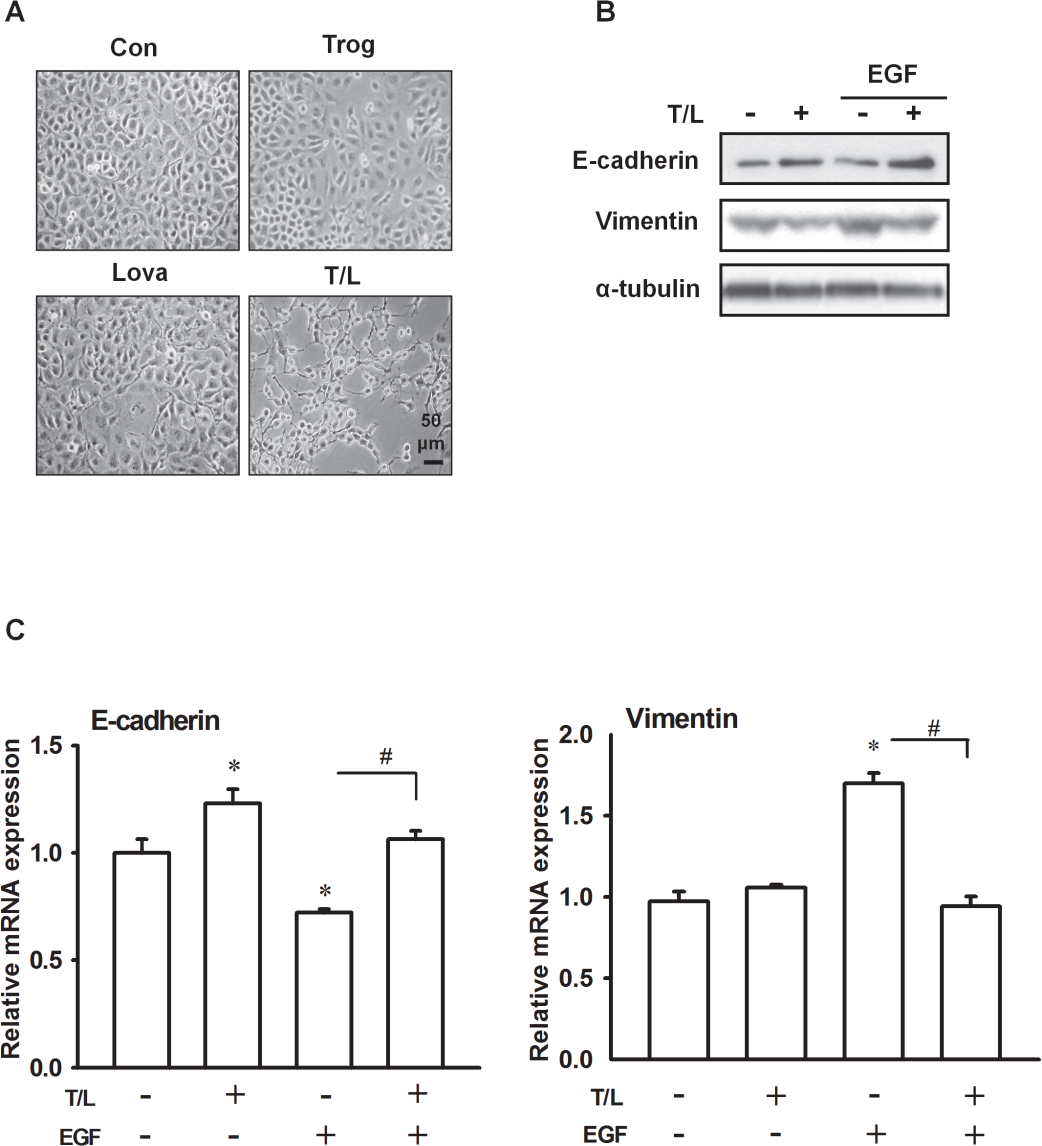
Effects of combined treatment with troglitazone and lovastatin on EGF-induced EMT-related marker protein expression in ATC cells. **A–C**. SW1736 cells were cotreated with troglitazone (5 μM) and lovastatin (1 μM) for 1 h and subsequently treated with EGF (10 ng/mL) for an additional 24 h. **A.** The cells were photographed, and representative pictures are shown. The (B) protein and (C) mRNA levels of E-cadherin and vimentin were determined by performing western blot analysis and real-time RT-PCR, respectively. All data are presented as the mean ± SE of 3 independent experiments. *, *p* < 0.05, compared with the control group; ^#^, *p* < 0.05. T/L, combined treatment with troglitazone and lovastatin.

Both membrane ruffling and filopodia formation are characterized as cytoskeletal remodeling and considered crucial for cell migration. Previous studies have indicated that the expression of epithelial-to-mesenchymal-transition (EMT)-related marker proteins, including the epithelial marker E-cadherin and the mesenchymal marker vimentin, changes during cell migration. We evaluated the combined effects of troglitazone and lovastatin on EGF-induced cytoskeletal organization and EMT-related marker protein expression in the SW1736 cells and observed that combined treatment with troglitazone and lovastatin inhibited EGF-induced membrane ruffling and filopodia formation in the SW1736 cells (Fig. 3). Neither troglitazone nor lovastatin significantly changed the cell morphology (i.e., spindle-like mesenchymal shape); however, combined treatment with troglitazone and lovastatin reverted the cell morphology to a rounded epithelial shape (Fig. 4A). Moreover, the cells subjected to the combined treatment exhibited a marked increase and decrease in the protein and mRNA levels of E-cadherin and vimentin, respectively (Fig. 4B and 4C). These results suggested that combined treatment with troglitazone and lovastatin can inhibit EGF-induced cytoskeleton reorganization and EGF-induced EMT-related marker gene expression.

### Combined Treatment with Troglitazone and Lovastatin Inhibited EGF-Induced Cyr61 Expression through the Downregulation of ERK/CREB Signaling Pathways

**Fig 5:**
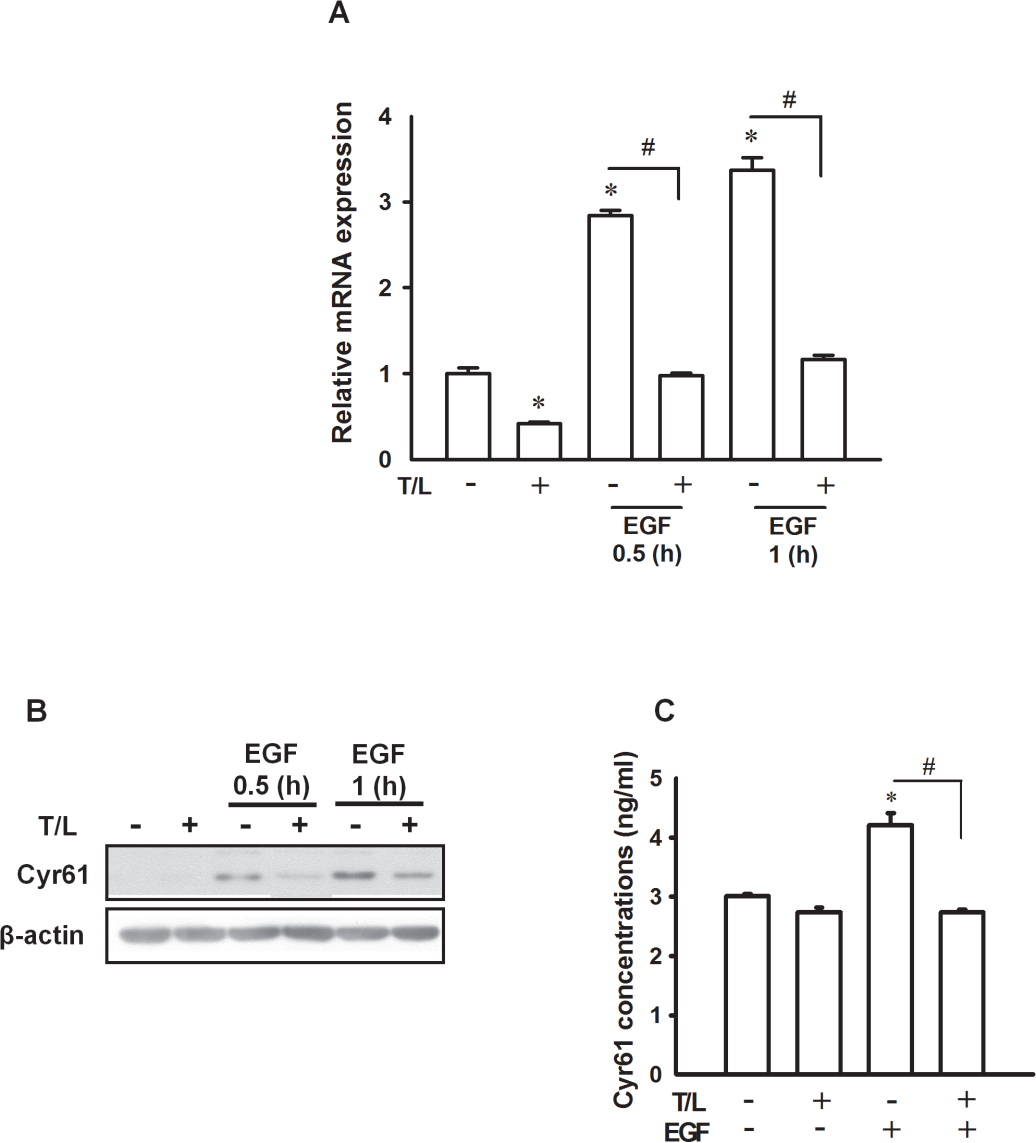
Effects of combined treatment with troglitazone and lovastatin on EGF-induced Cyr61 expression in ATC cells. **A–B**. SW1736 cells were cotreated with troglitazone (5 μM) and lovastatin (1 μM) for 4 h and subsequently treated with EGF (10 ng/mL) for the indicated duration. **A.** Total RNA was collected for detecting the mRNA levels of Cyr61 by using real-time RT-PCR, and **B.** the total cellular protein was collected to detect the protein levels of Cyr61 by performing western blot analysis. **C.** The SW1736 cells were cotreated with troglitazone (5 μM) and lovastatin (1 μM) for 0.5 h and subsequently treated with EGF (20 ng/mL) for an additional 1 h. The cultured medium was collected to determine the Cyr61 protein levels by using an ELISA kit. The data are presented as the mean ± SE of 3 independent experiments. *p < 0.05, compared with the control group. ^#^ *p* < 0.05. T/L, combined treatment with troglitazone and lovastatin.

Our previous studies have demonstrated that EGF-induced Cyr61 protein is vital for cell migration in ATC cells. We further examined whether combined treatment with troglitazone and lovastatin can inhibit EGF-induced Cyr61 expression in SW1736 cells. As shown in Fig. 5A and 5B, combined treatment with troglitazone and lovastatin suppressed in a time-dependent manner the mRNA and protein expression of Cyr61 in the EGF-treated cells. In addition, the cells subjected to this combined treatment exhibited decreased Cyr61 protein expression in the culture medium (Fig. 5C).

**Fig 6:**
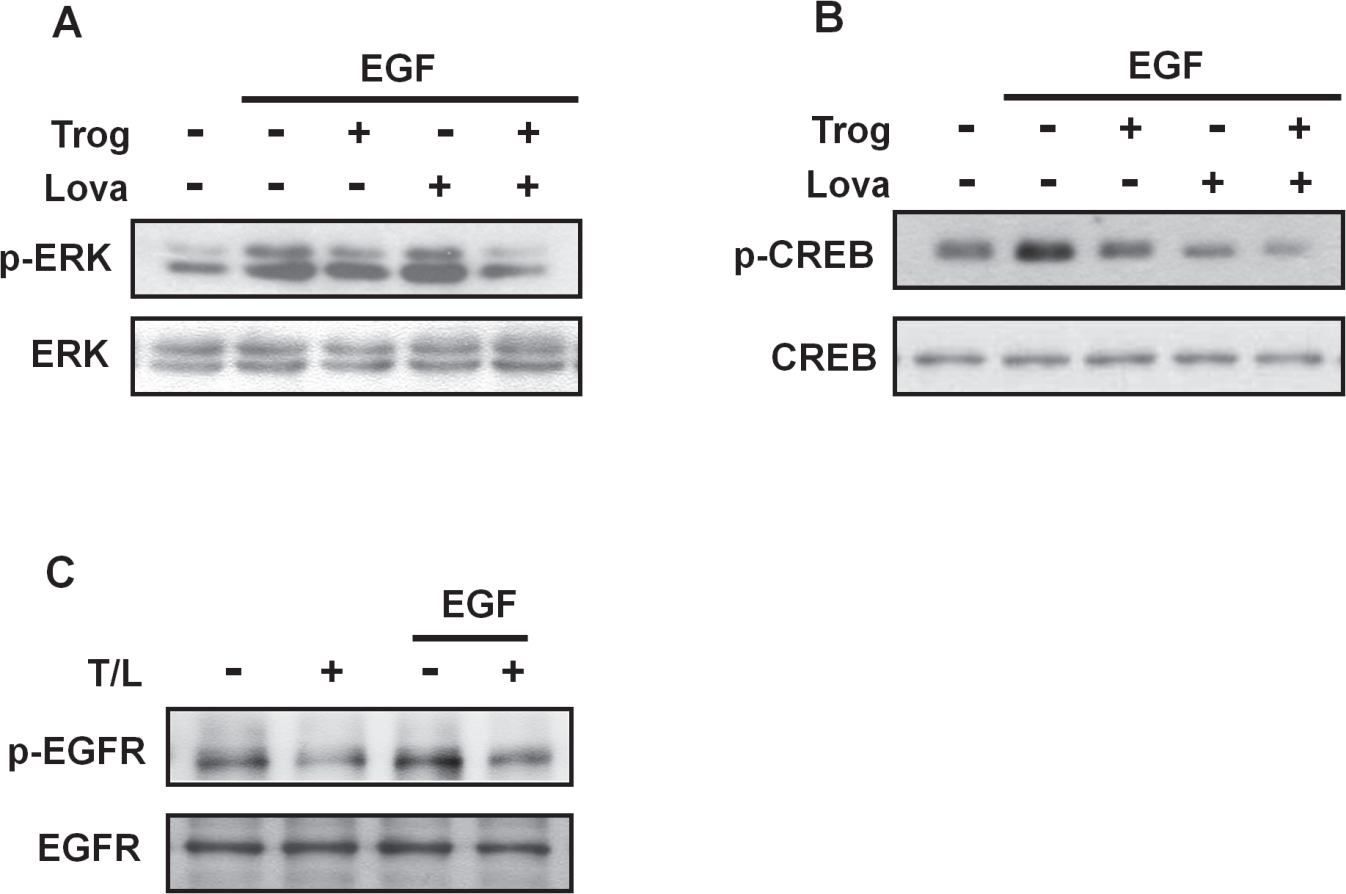
Effects of combined treatment with troglitazone and lovastatin on EGF-induced phosphorylation of ERK, CREB, and EGFR in ATC cells. **A–C.** SW1736 cells were treated with troglitazone (5 μM) and lovastatin (1 μM) for 4 h and subsequently treated wtih EGF (10 ng/mL) for (A) 15 min, (B) 30 min, or (C) 2 min. Total protein was collected to detect the phosphorylation levels of (A) ERK, (B) CREB, and (C) EGFR by performing western blot analysis. Trog, troglitazone; Lova, lovastatin; and T/L, combined treatment with troglitazone and lovastatin.

**Fig 7:**
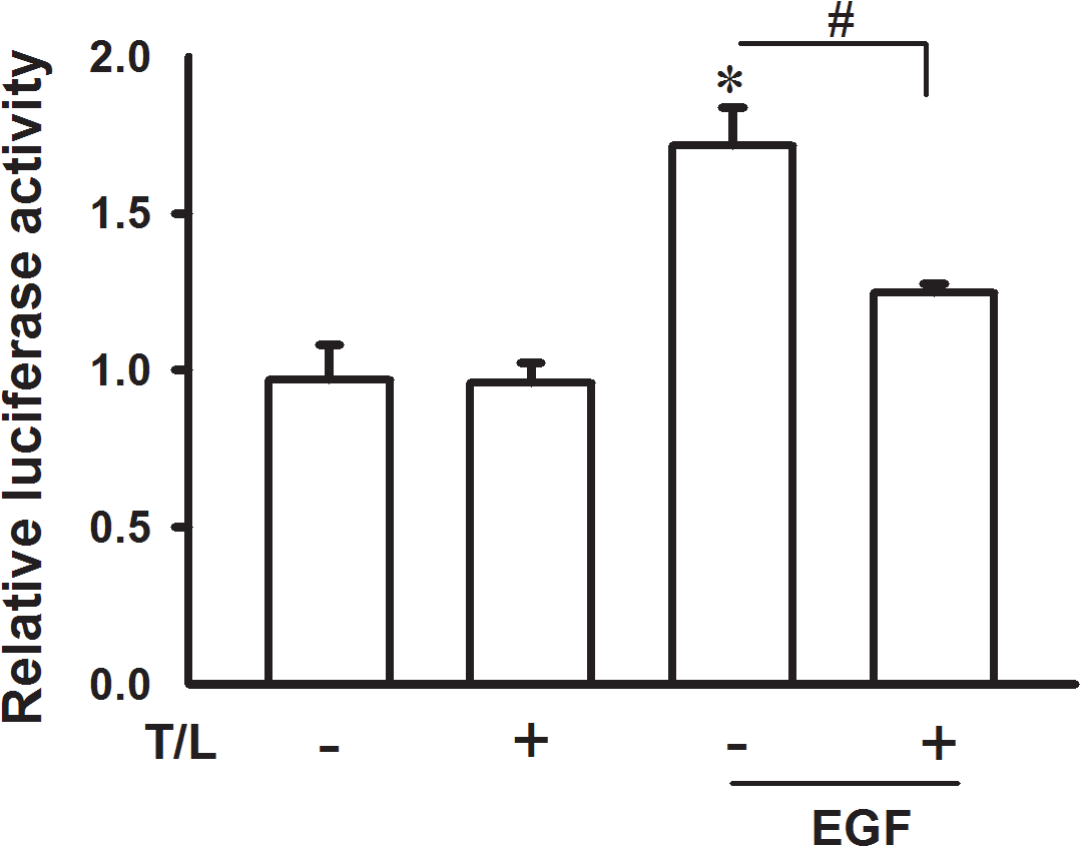
Effects of combined treatment with troglitazone and lovastatin on EGF-induced Cyr61 gene promoter activity in ATC cells. SW1736 cells were transfected with the Cyr61 reporter plasmid and phRL-TK plasmid as internal controls. The transfected SW1376 cells were then cotreated with troglitazone (5 μM) and lovastatin (1 μM) for 0.5 h and subsequently treated with EGF (10 ng/mL) for an additional 4 h. Total protein was collected to detect the Cyr61-promoter activity by using dual luciferase activity assay kits. The data are presented as the mean ± SE of 3 independent experiments. *, *p* < 0.05, compared with the control group; ^#^, *p* < 0.05. T/L, combined treatment with troglitazone and lovastatin.

Our previous studies have also indicated that EGF-induced Cyr61 expression is primarily mediated through the EGFR/ERK/CREB signaling pathways and several CREB-binding elements (CREs) that exist in the Cyr61 gene promoter. Therefore, we examined whether the combined treatment can inhibit EGFR, ERK, and CREB phosphorylation in EGF-treated cells as well as Cyr61-promoter reporter activity. As shown in Fig. 6, the combined treatment significantly inhibited the EGF-induced phosphorylation of the EGFR, ERK, and CREB as well as EGF-induced Cyr61-promoter activity (Fig. 7). These results suggested that the combined treatment inhibited cell migration by suppressing the EGFR/ERK/CREB signaling pathways as well as Cyr61 expression.

## Discussion and Conclusion

Our study and several other studies have demonstrated that statins, including lovastatin and simvastatin, can inhibit cell adhesion, migration, and invasion [18, 20, 21]. Our previous studies have revealed that EGF can induce Cyr61 expression through the activation of the EGFR/ERK/CREB signaling pathway and that EGF-induced Cyr61 is involved in the regulation of cell migration in ATC cells. In this study, low-dose lovastatin was combined with troglitazone to increase the antimigration activity of lovastatin. The results suggested that combined treatment with lovastatin and troglitazone markedly inhibited cell migration by inhibiting EGF-induced Cyr61 expression in ATC cells. This study is the first to demonstrate that combined treatment with low doses of troglitazone and lovastatin may be a useful strategy for treating ATC.

Lovastatin has been used to treat cancer in various animal experiments and clinical trials; however, the examined blood samples contained low concentrations of lovastatin. For example, in an animal model, the lovastatin concentrations observed in the tumor ranged between 0.023 and 0.41 μM after the administration of 50 mg/kg of lovastatin [22]. Holstein et al. reported that the peak lovastatin concentrations in patients’ blood ranged from 0.06 to 12.3 μM [23]. However, only 2 patients exhibited concentrations over 5 μM, with no antitumor responses. In addition, a mean concentration of only 3.9 μM was recorded in a trial conducted by Thibault [24]. These results indicate the inability of these statins to treat cancer when administered as a single agent. An in vitro study demonstrated that lovastatin and other statins may confer greater cytotoxic effects when combined with other anticancer drugs in cultured cells [23]. The growth-inhibitory effect of lovastatin on prostate cancer cells increased synergistically when combined with troglitazone [25]. This study demonstrated that combined treatment with plasma-achievable concentrations of troglitazone (5 μM) and lovastatin (1 μM) exerted significant antimigration effects on ATC cells.

**Fig 8:**
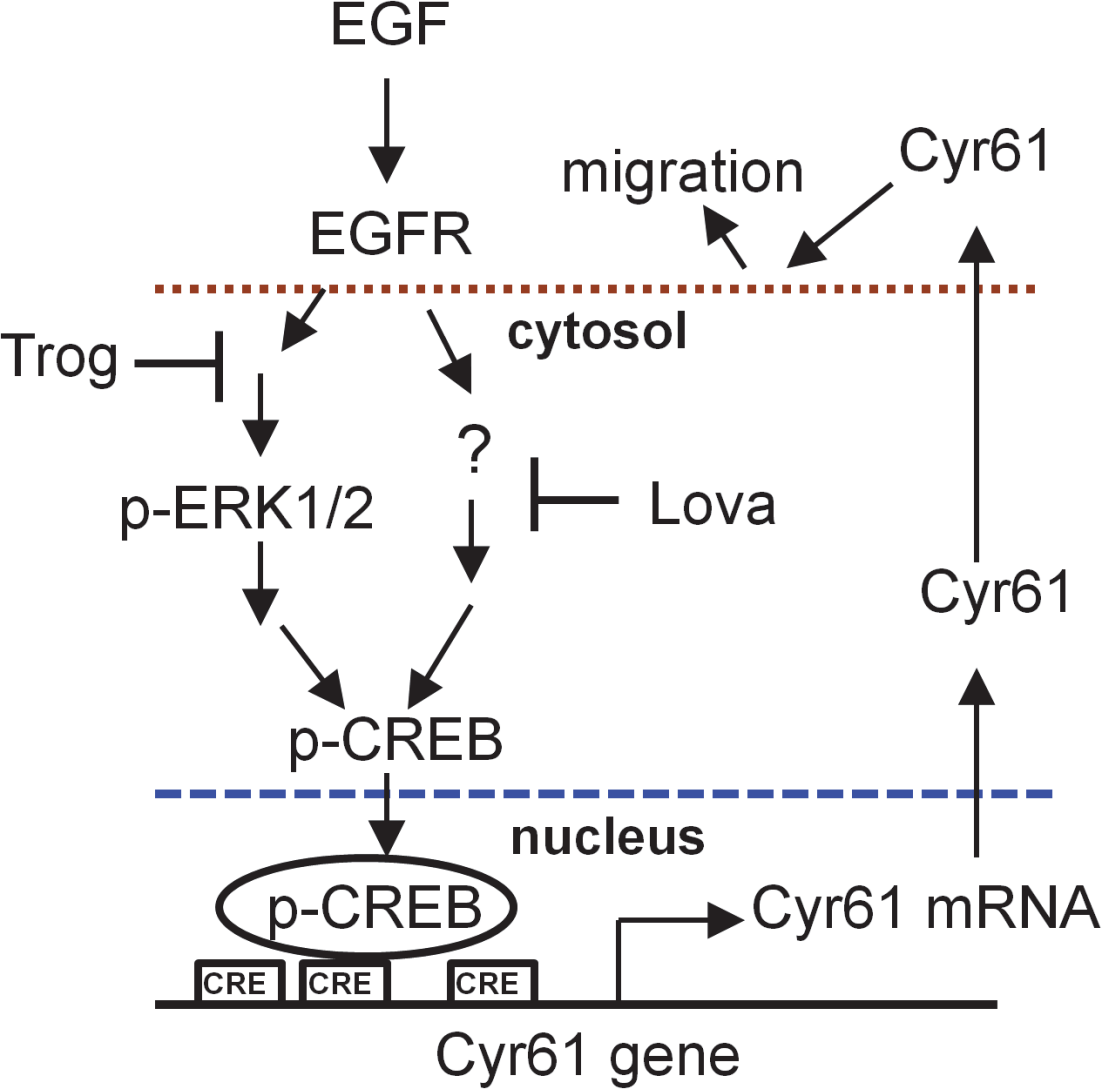
Proposed model of the effects of combined treatment with troglitazone and lovastatin on the inhibition of EGF-induced cell migration in ATC cells. EGF treatment increases Cyr61 expression through the ERK/CREB signal pathways and then promotes cell migration. Combined treatment with troglitazone and lovastatin downregulates CREB activity by inhibiting the ERK and other unknown signaling pathways, eventually reducing Cyr61 expression and cell migration in ATC cells. Trog, troglitazone; Lova, lovastatin.

Troglitazone and lovastatin regulate cell functions through various signaling pathways. Reportedly, troglitazone inhibited cell migration in human breast cancer cells by reducing focal adhesion kinase/Src phosphorylation [15]. In addition, troglitazone inhibited the proliferation of human prostate cancer cells by inducing ERK phosphorylation and PPARγ-independent pathways [26]. By contrast, troglitazone-inhibited ERK phosphorylation contributed to growth inhibition in human pancreatic cancer cells [27]. The present study revealed that troglitazone alone or combined with lovastatin can inhibit ERK phosphorylation in ATC cells (Fig. 6A). In addition, troglitazone did not activate PPARγ in the ATC cells, as determined using the PPARγ-reporter plasmid assay (data not shown). These results suggest that the antimigration activity of troglitazone may be mediated through ERK-dependent and PPARγ-independent pathways in ATC cells (Fig. 8). CREB is a transcription factor and can be activated through various signaling pathways, including the ERK, Ca^2+^, p90 ribosomal S6 kinase (p90RSK), mitogen- and stress-activated protein kinase (MSK), Ca^2+^/calmodulin-dependent protein kinase (CaMK), and MAPK-activated protein kinase 2 (MAPKAPK-2) pathways as well as other stress signaling pathways [28–30]. When activated, the EGFR can eventually phosphorylate and activate CREB through the ERK/p90RSK, ERK/MSK, or phospholipase C/CaMK signaling pathways. These findings showed that lovastatin can inhibit the phosphorylation of CREB but not that of ERK (Fig. 5), indicating that lovastatin may inhibit the phospholipase C/CaMK signaling pathway. A previous study indicated that lovastatin inhibited EGFR dimerization in squamous cell carcinoma cells [31]. We observed that combined treatment with troglitazone and lovastatin suppressed the autophosphorylation of the EGFR (Fig. 6C). These results suggested that the combined treatment may block EGF binding to the EGFR or EGFR dimerization, subsequently resulting in the inhibition of downstream CREB activation.

CREs exist in the proximal promoter region of the human Cyr61 gene [32], suggesting that CREB can regulate Cyr61 gene expression. However, the function of CREB in regulating Cyr61 gene expression is likely tissue specific. In melanoma, cDNA microarray analysis revealed that CREB silencing increased Cyr61 expression [33]. Conversely, CREB upregulated Cyr61 gene expression in sphingosine-stimulated human smooth muscle and osteoblastic cells [34, 35]. Reportedly, human Cyr61-promoter activation was regulated by CREB and activator protein-1 (AP-1) in sphingosine-stimulated human smooth muscle cells [34]. An AP-1 binding site identified in the human Cyr61 gene promoter was involved in the activation of the Cyr61 gene in response to hypoxia [36, 37]. AP-1 is a heterodimeric protein that comprises several protein families including c-Fos and c-Jun. c-Fos can be phosphorylated by ERK kinase in response to extracellular stimuli, increasing its transcriptional activity. In this study, combined treatment with troglitazone and lovastatin inhibited the ERK and CREB phosphorylation induced by EGF in ATC cells. These results indicated the involvement of both CREB and AP-1 in the regulation of Cyr61 gene expression in ATC cells cotreated with troglitazone and lovastatin.

Cyr61 can bind to the integrin α_v_β_3_ to promote cell adhesion, migration, and tubule formation [38]. Our previous study indicated that a Cyr61-neutralizing antibody completely inhibited EGF-induced cell migration. Combined treatment with troglitazone and lovastatin suppressed EGF-induced Cyr61 expression. These results suggest that the inhibition of EGF-induced migration by cotreatment with troglitazone and lovastatin may be mediated through the downregulation of Cyr61 expression. These results may provide a useful future strategy for treating patients with ATC.

## Supporting Information

S1 Fig Effects of combined treatment with troglitazone and lovastatin on EGF-induced cell migration in 8305C cells. The cells were preincubated with troglitazone (5 μM) and lovastatin (1 μM) for 1 h and then cultured in the presence or absence of 10 ng/mL EGF for 12 h. Cell migration was detected by using a wound healing assay. The data are presented as the mean ± S.E. of 3 independent experiments. *, *p* < 0.05, compared with the control group; ^#^, *p* < 0.05.
(TIF)
