# Correction: Combined Treatment with Troglitazone and Lovastatin Inhibited Epidermal Growth Factor-Induced Migration through the Downregulation of Cysteine-Rich Protein 61 in Human Anaplastic Thyroid Cancer Cells

**DOI:** 10.1371/journal.pone.0177545

**Published:** 2017-05-08

**Authors:** Li-Han Chin, Sung-Po Hsu, Wen-Bin Zhong, Yu-Chih Liang

There is an error in affiliation 2 for authors Sung-Po Hsu and Wen-Bin Zhong. Affiliation 2 should be: Department of Physiology, School of Medicine, College of Medicine, Taipei Medical University, Taipei, Taiwan.
