# Supplementary figures and images for: Combined Treatment with Troglitazone and Lovastatin Inhibited Epidermal Growth Factor-Induced Migration through the Downregulation of Cysteine-Rich Protein 61 in Human Anaplastic Thyroid Cancer Cells
Source: PLoS One. 2015 Mar 5;10(3):e0118674. doi: 10.1371/journal.pone.0118674 (PMC4351011; doi:10.1371/journal.pone.0118674)

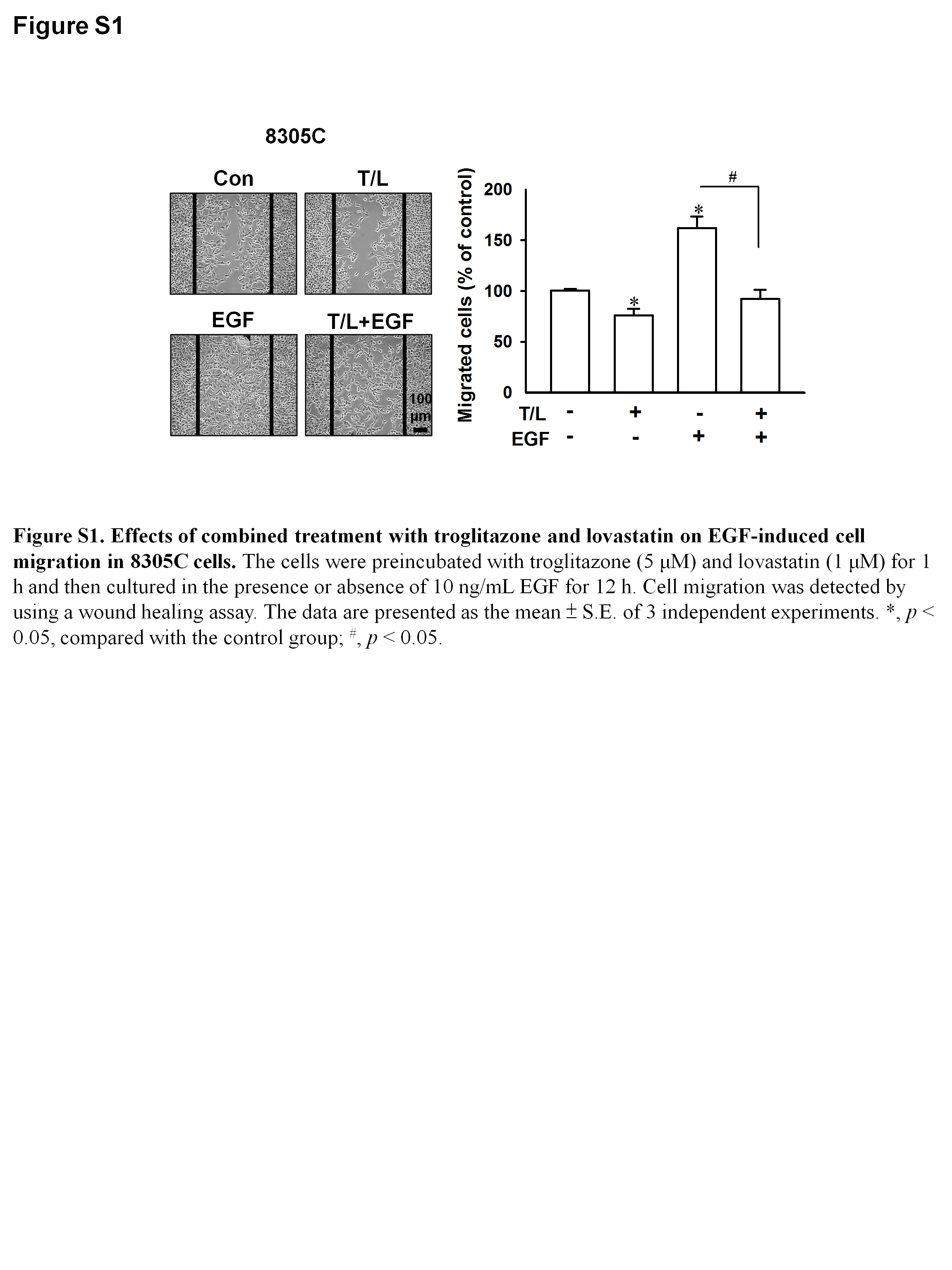

Supplement: S1 Fig — The cells were preincubated with troglitazone (5 μM) and lovastatin (1 μM) for 1 h and then cultured in the presence or absence of 10 ng/mL EGF for 12 h. Cell migration was detected by using a wound healing assay. The data are presented as the mean ± S.E. of 3 independent experiments. *, p < 0.05, compared with the control group; #, p < 0.05. (TIF) [file pone.0118674.s001.TIF]
